# Rethinking Causation in Cancer with Evolutionary Developmental Biology

**DOI:** 10.1007/s13752-018-0303-0

**Published:** 2018-07-27

**Authors:** Katherine E. Liu

**Affiliations:** Department of Ecology, Evolution, and Behavior, University of Minnesota, Saint Paul, MN USA

**Keywords:** Biomarker, Cancer, Causation, Evolutionary developmental biology, Evolutionary medicine, Evolvability, Modularity, Proximate–ultimate

## Abstract

Despite the productivity of basic cancer research, cancer continues to be a health burden to society because this research has not yielded corresponding clinical applications. Many proposed solutions to this dilemma have revolved around implementing organizational and policy changes related to cancer research. Here I argue for a different solution: a new conceptualization of causation in cancer. Neither the standard molecular biomarker approaches nor evolutionary biology approaches to cancer fully capture its complex causal dynamics, even when considered jointly. These approaches map on to Ernst Mayr’s proximate–ultimate distinction, which is an inadequate conceptualization of causation in biological systems and makes it difficult to connect developmental and evolutionary viewpoints. I propose looking to evolutionary developmental biology (EvoDevo) to overcome the distinction and integrate the proximate and ultimate causal frameworks. I use the concepts of modularity and evolvability to show how an EvoDevo perspective can be manifested in cancer translational research. This perspective on causation in cancer is better suited for integrating the complexity of current empirical results and can facilitate novel developments in the investigation and clinical treatment of cancer.

## Introduction

Cancer research has made tremendous strides in recent decades, but it has yet to make the progress that we need it to. That is, numerous cancer-related reports are published each week, yet the rate at which discoveries are translated into effective clinical applications is low. Various parties have pointed to different reasons for this situation, including funding and institutional barriers to research, as well as a conservative system that is fairly unsupportive of physicians deviating from the norm. However, translation failure rates remained high despite a twofold increase in the US National Institutes of Health (NIH) budget between 1998 and 2003 (Sarewitz 2013). The NIH, the Canadian Institutes of Health Research (CIHR), and other funding organizations have recently emphasized translational research: an endeavor specifically focused on translating the knowledge gained at the bench into clinical applications. Much of the attention in translational research has been devoted to organizational and policy changes that remove barriers to clinical trials, redesigning physician-scientist (MD/PhD) programs, and facilitating programs that encourage the formation of academic centers for interdisciplinary clinical and translational research which include members from basic research, biomedical research, industry, and practicing physicians, among others (e.g., the Clinical Translational Science Awards consortium through the NIH). However, less attention has been paid to whether the core research strategies used in translational research are adequate for the generation of desired clinical applications. It is assumed that prevailing approaches and frameworks will translate into clinical applications eventually, given enough time and the right combination of researchers. But this is a bad assumption; attending to institutional structure and research organization alone will not be sufficient. Cancer translational research needs a more radical solution.

I am not the first to recognize this, nor the first to suggest a solution to this problem. Evolutionary and ecological approaches to cancer research emerged in response to the lack of clinical applications from the basic biology discoveries (Wodarz 2006; Greaves and Maley 2012; see more below), and the parallels between cancers and ecosystems continue to be elucidated (Merlo et al. 2006; Kareva 2015). Physicists and mathematicians were recruited by the US National Cancer Institute to ask questions about cancer in new ways, including using their mathematical and computational skills to model and investigate virtual cancer systems (Michor et al. 2011; Agus and Michor 2012; Kuhn and Nagahara 2013). Philosophers also have sought to explain and make sense of cancers in novel ways (Bertolaso 2016), such as through multilevel selection (Lean and Plutynski 2015) or cancer stem cell theory (Laplane 2016). Although these suggestions have led to valuable research programs, they miss a key issue at the root of the difficulties facing cancer translational research: the conceptualization of causation in cancer.

The central thesis of this article is that cancer translational research needs a new perspective on causation. In the next section, I review two predominant approaches to investigating cancer (the molecular biomarker approach and the evolutionary biology approach), and dissect their causal frameworks. Then in the following section, I argue that the frameworks are insufficient as they appear to map on to Ernst Mayr’s proximate–ultimate distinction, which is an inadequate conceptualization of causation in biological systems. In the fourth section, I suggest that this problem can be solved by looking at evolutionary developmental biology, which is an integration of ultimate causes (from evolutionary biology) and proximate causes (from developmental biology). I draw on discussions around major concepts in evolutionary developmental biology, specifically modularity and evolvability, and apply those concepts to cancer translational research. I make some concluding statements in the last section.

Before proceeding, I want to make explicit how I understand the notions of “causation” and “causality.” I use the terms “causality,” “causes,” and “causation” nonoperationally. That is, my goal is not to quantify causes or causal input, nor is it to identify the main cause(s) of cancer. Here, I want to suggest a novel conceptualization of how various causes are related. Once we are satisfied with the conceptualization of causation, the next step is to operationalize that conceptualization for the detection of causes. However, this step is outside the scope of this article.

## Predominant Approaches and Frameworks for Investigating Cancer

### Molecular Biomarker Approach

Biomedical research has become increasingly molecular with the availability of technologies such as whole genome sequencing. The promise has been that a better understanding of molecular mechanisms will make targeted clinical interventions possible. These interventions modify or correct abnormalities in particular individuals while minimizing side effects that come with traditional blanket treatments such as chemotherapy. The focus on targeted interventions in individuals has fueled the rise of precision medicine^1^ (Collins and Varmus 2015). One version that has become widespread is based on sequencing the genomes of individuals. The goals for this project are to develop and identify drugs that will be most effective based on the patient’s specific genomic composition. Because genomes can be sequenced, scanned, and compared quickly and (relatively) inexpensively, researchers are especially interested in identifying variants that are biological markers of diseases (biomarkers). These variants are supposed to be differences between healthy and sick individuals that give insight into how patients will react to drugs, which allows for tailored prescriptions and dosages.

One of the most successful examples of the molecular biomarker approach is trastuzumab, also known as Herceptin (Genentech).^2^ The FDA approved trastuzumab for the treatment of metastatic breast cancer in 1998 (Ignatiadis et al. 2009), and it has been heralded as having a profound impact on the care of HER2+ breast cancers^3^ (Hudis 2007), and as “a major advance in targeted cancer therapies” (National Breast Cancer Coalition 2013). Trastuzumab is a humanized monoclonal antibody with two binding sites specific to the extracellular portion of HER2, a cell surface protein associated with the formation of homodimers as part of a cell growth signal pathway. When HER2 is overexpressed, the homodimers continually signal cells to grow and divide. Trastuzumab binds to the HER2 protein and blocks dimerization, which prevents further activation of signaling. Additionally, the antibody triggers the immune system to target HER2+ cells with increased antibody-dependent cell-mediated cytotoxicity (Hudis 2007).

This example shows how the molecular biomarker framework can work. Researchers identified a mechanism (a cell growth signaling pathway), which is causally relevant to carcinogenesis, and a specific biomarker (the HER2 protein) where a targeted intervention should substantially make a difference. Original clinical trials with HER2+ metastatic breast cancer patients showed positive results in 12–25% of the cases, depending on previous treatment regimes, thus providing a proof of principle for the effectiveness of trastuzumab. In later trials, when administered in an adjuvant setting (i.e., trastuzumab plus chemotherapy), the risk of recurrence dropped by 40–50% and the risk of death dropped by one-third (Ignatiadis et al. 2009). In comparison to most cancer treatments, these results are impressive. However, given the straightforward nature of the intervention, greater response rates were expected. Additionally, further analyses suggest that the chemotherapy accounts for much of the efficacy (Moja et al. 2012). Some anticipate that identifying more tumor characteristics (including more biomarkers) will lead to better efficacy (Ignatiadis et al. 2009). Others have suggested that the limited efficacy is due to misplaced attention on HER2 expression. That is, HER2 expression is a bad proxy for identifying which subpopulations will benefit from this drug. Michalis Karamouzis and colleagues (2007) suggested that heregulin, a ligand that binds to multiple receptors in the cell growth signal pathway just described, might be a better biomarker than HER2 overexpression.

The fact that trastuzumab was developed to treat invasive or metastatic cancers but is shown to be effective only in early stage cancers^4^ says something significant about the causality of cancer. The molecular biomarker approach might work if its target was part of a relatively static system, but diseases like cancer are dynamic and progressive. Molecular biomarker approaches ignore the developmental nature of cancer in individuals, and of disease biology more generally. Disease-affected systems in individuals are complex and have evolved robust and redundant pathways in order to address circumstances of disturbance. To investigate and understand these complex systems, multilevel and integrated approaches are needed to account for causal dynamics involving feedback loops and robustness at different levels and across different timescales (Mitchell 2009). The molecular biomarker approach alone is insufficient.

## Molecular Biomarkers as Difference Makers

In order to see why the molecular biomarker approach is insufficient, it is important to look at the causal framework employed. Recall that this approach involves finding the molecular differences between healthy and unhealthy individuals (or healthy and unhealthy tissues) and then intervening on those differences to change the resulting phenotype. Thus, the molecular biomarker is what makes the difference between being healthy and unhealthy.

Many experiments designed to understand causal structures are modeled on methods following John Stuart Mill’s methods of difference: if you have two identical systems differing in only one factor, then differences in outcome can be attributed to that differing factor. Under these conditions, the factor is a “difference maker.” Knockout experiments are a widely used form of this causal reasoning strategy. For example, to infer the role of the gene *TP53* in cancer, one can compare standardized mice, some with an inactivated form of *TP53* and some with functioning *TP53*. If mice with inactivated *TP53* show abnormal cell development that results in tumors, whereas mice with functioning *TP53* show normal cell development, then—assuming all other factors are equal^5^—we can infer that the *TP53* gene plays a causal role in cell development (Mitchell 2009).

Translational research, though, is not just about identifying differences. Because the overall goal of translation is successful treatment, intervention resulting in a specific outcome is key. Thus researchers want to be able to identify the biomarker and then be able to change it such that the resulting phenotype is the desired one. James Woodward (2010) has an interventionist account of causation that formally describes the casual framework of the molecular biomarker approach:

*X* causes *Y* if and only if there are background circumstances *B* such that if some (single) intervention that changes the value of *X* (and no other variable) were to occur in *B*, then *Y* or the probability distribution of *Y* would change. (p. 290)

This framework can be used in two ways: to identify causes and justify interventions. For the former, we can look back on the knockout experiment previously described. Using standardized mice is a way to achieve the same background circumstances (*B*). The variable *X* takes two values: functioning *TP53* and inactivated *TP53*. The variable *Y* takes on two values as well: normal cell development and abnormal cell development. This gives the following causal formulation: if one intervenes on *TP53* and changes its value from functioning to inactivated, and the value of the normal cell development changes to abnormal, then one can infer that *TP53* causes (i.e., makes a difference to) normal cell development.

Alternatively, we can start with statements about known causes such as “Inactivated *TP53* causes abnormal cell development” and “Functioning *TP53* causes normal cell development.” Then using Woodward’s framework, translational researchers should be able to say, “Given that functioning *TP53* causes normal cell development, with background circumstances B present, changing the value of *TP53* from inactivated to functioning will change the value of cell development from abnormal to normal.” Thus clinicians will be able to identify and justify where to intervene to get the result they want.

This example requires changing a gene, which is not the usual or desired approach. Ideally, researchers want to intervene on components of pathways. To return to the trastuzumab example, we can consider X to have the values of homodimers present and absent, and Y has the values of cells growing and dividing or not. Thus, theory says that if they develop a protein that changes the value of X from homodimers present to absent, then Y will change from cells growing and dividing to not.

Despite the value of this causal framework, there are several limiting assumptions related to its application to cancer translational research.^6^ The first assumption is that there is a one-to-one relationship between the variant and the disease phenotype; i.e., “*X* causes *Y*,” not “*X* and *Z* cause *Y*” or “*X* causes *Y* and *W.”* Many-to-one relationships (“*X* and *Z* cause *Y*”), either as multiple pathways resulting in the same phenotype or many causal factors contributing to one phenotype, can make it difficult to know where to intervene because these cases facilitate redundancy in the system that, in some cases, compensates for the intervention. Additionally, interventions on one-to-many or many-to-many relationships (“*X* causes *Y* and *W*” or “*X* and *Z* cause *Y* and *W*”), such as genes showing pleiotropic effects in two or more seemingly unrelated pathways or phenotypes, can lead to unwanted outcomes (side effects). In other words, it is difficult to change *X* without affecting other variables. Thus, redundant pathways and pleiotropy will constrain the success of the difference-making causal framework used in the molecular biomarker approach. Even if the patient was prescribed two treatments (*X* causes *Y* and *Z* causes *Y* so treatment1 changes *X* to X* and treatment2 changes *Z* to Z*), changing the other value changes the background conditions so now the causal claims do not necessarily hold.

A second assumption is that the background conditions remain the same when you change the value of the variable (e.g., from functioning *TP53* to inactivated *TP53*). That is, to test if functioning *TP53* makes a difference on normal cell development, one needs a group of individuals with functioning *TP53* and a group of individuals with inactivated *TP53*. Additionally, for this causal framework to apply, the activation of *TP53* has to be the only difference. This might be possible (or close to possible) in laboratory conditions with the use of standardized model organisms or cell cultures, but in the clinical setting this assumption does not hold. The same mutation can be malignant in one individual but not in another depending on the genomic background or previous mutations (Greaves and Maley 2012). As well, this assumption makes it difficult to identify causal variables if the researchers are using clinical data rather than laboratory conditions. The variation from one individual to another often violates the assumption that the background conditions are similar, and the partial effectiveness of trastuzumab in treating breast cancer illustrates this clearly.

Finally, this framework tends to treat all difference-making factors symmetrically. That is, it assumes that all difference-makers in that system will affect the outcome in similar ways, such that there is no principled way to choose which factor(s) to privilege. Woodward (2010) suggests that knowledge of other characteristics of causal relationships, such as stability and specificity, will lead to more nuanced pictures of causal relationships, though the conditions for stability and specificity are less precisely characterized than those for difference-making. Interventions relevant to the clinical treatment of cancer (e.g., via surgery or chemotherapy) require not only that a factor be identified as an actual difference-maker (versus a potential difference-maker); they also require detailed knowledge of *how* it makes a difference (e.g., how stable or specific the relationships are). Thus, identifying actual difference-makers permits the identification of multiple causes relevant to tumor progression or other aspects of cancer (Waters 2007), but it does not, on its own, suggest further characteristics that would facilitate successful clinical applications.

It is important to note that cancer researchers may or may not think that these three assumptions (there are one-to-one causal relationships, background conditions are sufficiently similar, and adequacy of difference-makers) are appropriate or true of the world. The point is that they are assumptions of the causal framework used to identify molecular biomarkers. Regardless of whether researchers share each of the assumptions of the causal framework, they are embedded in the molecular biomarker approach. These methodological assumptions help to explain why this approach is insufficient to dissect cancer’s complex causal dynamics.

### Evolutionary Biology Approach

Towards the end of the 20th century, a group of clinicians joined forces with a group of evolutionary biologists to reconnect medicine and evolutionary biology for what they termed Darwinian medicine. Although a driving aspect of this enterprise was the incorporation of evolutionary biology into medical school curricula (Williams and Nesse 1991; Nesse and Schiffman 2003; Nesse 2008; Nesse et al. 2010), the main research goals were to derive new insights into both medical and evolutionary questions, as well as to change clinical practices (Williams and Nesse 1991; Nesse 2001; Nesse and Stearns 2008; Stearns 2012; Valles 2012). The types of questions asked are: can an evolutionary perspective explain why particular symptoms appear? Why are certain individuals more susceptible to certain diseases? How should answers to the questions modify patient care? (Nesse 2001; Nesse et al. 2006, 2010; Nesse and Stearns 2008).

Darwinian medicine has answered many medical questions utilizing population genetic theory and an adaptationist research program (Nesse 2008; Nesse and Stearns 2008; Valles 2012). An important example is explaining the prevalence of sickle cell anemia through heterozygote advantage. Individuals that are homozygous for the recessive sickle cell allele (SS) suffer from sickle cell anemia, which is usually fatal due to the formation of blood clots and decreased oxygen delivery to tissues. Individuals that are homozygous for the dominant allele (AA) have normal red blood cells but are susceptible to malaria. Those that are heterozygous (AS) have both normal and sickled red blood cells, and have increased resistance to malaria, as the sickled phenotype makes the red blood cells less suitable for the malaria-causing parasite. In environments where malaria is epidemic (e.g., many regions of Africa), the AS genotype is more fit than either the AA or the SS genotypes. Therefore, under these environmental conditions, the S allele will be maintained in the population and thus sickle cell anemia will continue to persist. Note that this explains the persistence of sickle cell anemia but does not suggest any changes to clinical practices.

Antibiotic resistance, on the other hand, is a common example used to demonstrate medically relevant evolution in real time (Stearns 2012) and also has potential for clinical application. Resistance was long thought to be a result of evolved coexistence with the host. But selection leads to higher virulence of pathogens, in part due to rapid parasite evolution (Williams and Nesse 1991; Nesse 2008; Nesse and Stearns 2008), and thus clinical efforts to minimize resistance were previously misinformed. By administering large doses of antibiotics to reduce the chance of de novo resistance mutations occurring, clinicians were actually selecting for rare resistance genes already present in the population, which could then be horizontally transferred to otherwise susceptible bacteria. This has led to more discussions about the use of antibiotics in hospitals and clinics, including how to balance the killing of bacteria of an infection without strongly selecting for existing drug-resistant bacteria (Smith et al. 2015).

A key contrast between the molecular biomarker approach and the Darwinian medicine approach is that the former concerns individuals and the latter populations. However, the examples of sickle cell anemia and antibiotic resistance show that the appropriate level at which to consider “the population” in evolutionary medicine can vary from populations of people (e.g., in the sickle cell anemia case) to populations of cells (or populations of microbes in the antibiotic resistance case). With respect to cancer, it is no different. For populations of people, mismatches between past and current environmental contexts can explain the persistence of vulnerabilities to cancer (Greaves 2002; Aktipis and Nesse 2013). Additionally, population genetics can be applied to cancer incidence data to test hypotheses about the causal roles of mutation and drift in cancer (Frank 2007). This informs predictions about what factors are connected with shifts in incidence curves (measurements of how cancer occurrence varies with age), and thus when it is important to start screening for particular cancers in certain populations.

With respect to populations of cells, Peter Nowell proposed a model of clonal evolution in tumors in which they develop over time through a series of stepwise somatic mutations followed by repeated selection (Nowell 1976). A mutation occurs in a somatic cell (either spontaneously or induced by a carcinogen) that gives it a growth advantage over neighboring cells and facilitates the clonal expansion of cells containing that mutation. As the population of cells expands, more mutations accumulate, some of which will give additional selective advantages, allowing those subpopulations with the advantage to be selected for and to further expand. This process results in tumors that reflect specific and local environmental pressures. A more nuanced account of this process treats the tumor as a byproduct of the tissue ecosystem (Greaves and Maley 2012). Each time a treatment (e.g., chemotherapy) is given, it changes the ecosystem, which changes the development of the tumor. As a result, if part of the tumor is removed, then the tumor that recurs is a different tumor than what was originally there. This model illuminates why there is variation within a tumor and differences between individuals with the same kind of cancer.

A primary goal of evolutionary medicine is to have clinical impact. By analyzing incidence data from populations of people, we can develop screening policies. By applying evolutionary theory to populations of cells, new treatment regimens are suggested, such as adaptive therapy, where the goal is to maintain tumors at a stable size rather than fully eliminate all cancer cells^7^ (Gatenby et al. 2009; Enriquez-Navas et al. 2016). Cytotoxic drugs select for resistant cells and create an environment where the resistant cells can proliferate, causing a tumor to become more aggressive and harder to treat. Thus, with adaptive therapy, tumor status is continually assessed and treatment plans are adjusted to achieve a desired tumor population such that chemosensitive cells keep chemoresistant cells suppressed and limit their proliferation. That is, the dose of chemotherapy may increase, decrease, or not be administered at all, depending on the status of the tumor.

As with antibiotic resistance, investigating cancer through a Darwinian lens can lead to better uses of existing treatments. However, this approach yields little mechanistic information that is relevant for the development of new treatments (e.g., new drugs). More to the point, the approach does not help treat metastasis, the pathological form of cancer that is responsible for most deaths. The fact that a Darwinian medicine approach gives predictive power and thus offers preventive possibilities is incredibly important. Work done in this way suggests treating cancers early, presumably to never reach metastasis. However, not every case of cancer can be diagnosed early, nor should we necessarily depend on early detection and prevention (Narod et al. 2015; Prasad et al. 2015). Thus, we still lack effective approaches for the investigation and clinical treatment of cancer, especially metastatic cases. The Darwinian medicine approach alone is insufficient.

### Natural Selection and Population-Level Causes

As with the molecular biomarker approach, it is useful to look at the causal framework employed by the Darwinian medicine approach. Population genetics is the basis of Darwinian medicine (Nesse 2008; Nesse and Stearns 2008), which means natural selection, mutation, drift, and migration are key factors in its causal framework. However, in practice, Darwinian medicine prioritizes natural selection as a cause, making it an adaptationist program (Valles 2012). For example, its answer to why we are still vulnerable to certain diseases is that humans are “bundles of compromises shaped by natural selection… to maximize reproduction, not health” (Nesse and Stearns 2008, p. 28). That is, much of the research program focuses on maladaptations (Nesse 2005). Individual organisms in a population show heritable variation with fitness differences, but natural selection is a population-level cause (see, e.g., Millstein 2006). Consequently, the causal framework of Darwinian medicine does not speak to the condition of specific individuals in the population.^8^

Notably, mutation, drift, and migration play more prominent causal roles in evolutionary approaches to cancer than in traditional Darwinian medicine (Merlo et al. 2006; Aktipis and Nesse 2013). Because of this, I wish to separate evolutionary approaches to cancer from Darwinian medicine, which has traditionally prioritized natural selection. Mutations create the heterogeneity in a tumor allowing for faster progression and therapeutic resistance (Merlo and Maley 2010; Park et al. 2010; Aktipis and Nesse 2013). Genetic drift occurs when population sizes are small. While tumors are generally large populations of cells, metastasis (the spread of cancer to other locations in the body) occurs via small clusters of cells that create population bottlenecks (Aceto et al. 2014). Additionally, cancer stem cell populations are usually composed of only a few cells (Baker et al. 2014), which explains why deleterious mutations can go to fixation and why some tumors might be more aggressive than others. Migration plays several roles in cancer biology. One is related to mismatches between ancestral conditions and modern environments. For example, light-skinned individuals migrating towards the equator creates a mismatch between skin pigment and sun exposure, and thus changes their susceptibility to skin cancer (Aktipis and Nesse 2013). Migration might also be important in explaining metastasis, which inherently involves the migration of cells from one location to another. One model suggests that heterogeneity in a tumor selects for migratory cells (Chen et al. 2011).

Although these evolutionary insights into cancer complement what has been discovered in terms of molecular mechanisms, they have not yet had a clinical impact. They account for why it is important to catch and treat cancers early, but do not give mechanistic information that would be useful for treating existing cancers in specific individuals. As a consequence, evolutionary approaches alone are insufficient for dissecting cancer’s complex causal dynamics.

## Combining the Molecular Biomarker and Evolutionary Approaches?

Both the molecular biomarker and evolutionary medicine approaches give us important information about cancer biology, and so both approaches are necessary for making progress towards treating cancer. However, neither is sufficient to dissect the causally complex dynamics of cancer, even when considered jointly. The approaches ask fundamentally different questions and therefore investigate causes using different frameworks. Researchers using the molecular biomarker approach want to know *how* cancers arise and progress in individuals, and *how* specific molecules make a difference to the outcome or phenotype. The identification of and dependence on biomarkers is a move to more closely connect the biology of the specific cancer to a treatment. Thus, it becomes more important for researchers to know the biological role certain biomarkers have in cancers to best develop effective treatment plans (Henry and Hayes 2012; Thariani et al. 2012). Alternatively, those using the evolutionary biology approach want to know *why* cancers occur and progress, and *why* certain populations are more susceptible (Aktipis and Nesse 2013). The differences in how questions are formed between the biomarker and evolutionary biology approaches bear resemblance to Ernst Mayr’s proximate–ultimate distinction.

In a seminal paper, Mayr distinguished between two different kinds of biological causes: proximate and ultimate (Mayr 1961). Proximate causes are those studied by functional biologists (e.g., physiologists and developmental biologists) and often answer “How?” questions. Explanations that come from this orientation involve immediate factors, such as day length or hormone levels. For example, a change in hormone levels causes an organism to change its behavior. In contrast, Mayr characterized ultimate causes as those that involve evolutionary history and are present due to many generations of natural selection. These explanations are historical in nature and often answer “Why?” questions. For example, an organism has a behavior because it was beneficial to its ancestors and was selected for within a population over many generations. In this 1961 paper, Mayr aimed to differentiate proximate and ultimate causes while arguing that both kinds of causes are necessary for a complete understanding of any biological phenomenon.

In cancer biology, the overexpression of the HER2 protein in breast cancers can be considered a proximate cause. That is, overexpression of HER2 causes the formation of homodimers which continually signal the cells to grow and divide, thus causing cancerous growth. On the other hand, one might suggest that because natural selection maximizes reproductive success and not health, women with early menarche are more susceptible to breast cancers (Aktipis and Nesse 2013). That is, selection for reproductive success is an ultimate cause of breast cancers. Thus, according to Mayr, the molecular biomarker and evolutionary biology approaches might together yield a complete causal framework for understanding causation in cancer. Although having both approaches does give a fuller causal picture than just one approach alone, there are reasons why we might not want to interpret the biomarker and evolutionary biology approaches as separate or distinct in this way.

Laland and many of his colleagues have argued that the distinction between proximate and ultimate causes, though helpful at times (e.g., it prevents scientists from talking past each other, and allowed evolutionary biology and developmental biology to independently grow and mature as disciplines), has actually hindered progress in evolutionary biology (Laland et al. 2011, 2012). This is because the distinction has become entrenched as a truth about the nature of causation, rather than as a heuristic in the study of causes (Laland et al. 2012). It has shifted from being a good way to approach causality in biological systems to being the only way one can understand causation in biological systems. The mentality has become entrenched because Mayr’s basic premise—proximate and ultimate causes answer different questions and are not in competition with one another—implies that there is no reason to link them; proximate causation is not important to the investigation of ultimate causes and vice versa (Laland et al. 2012). This makes it difficult for the approaches to mutually inform one another in a way that would contribute to clinically relevant outcomes, where a more complete picture of the causal landscape is necessary. This mentality appears to be manifested in cancer research, where the two approaches are funded through different avenues and the research communities minimally overlap.^9^

Maintaining the proximate–ultimate distinction leads to other problems as well. First, Laland et al. (2011, 2012) argue that this distinction cannot account for cases of reciprocal causation. If developmental processes are interpreted as proximate causes and separated from evolutionary processes (ultimate causes), we miss the fact that developmental processes contribute to the phenotypes on which selection acts, and these mechanisms are under selection themselves. Thus, developmental processes (proximate causes) are both an input and an output of ultimate causes (e.g., selection). Second, the proximate–ultimate distinction and how-why distinction are false dichotomies because it is possible to ask questions that can be answered with both or neither category (Calcott 2013). Calcott also points out that considering ultimate questions to be historical explanations can be confusing, as it is not clear what it means for an explanation to be historical. This affects the range of possible answers to questions regarding ultimate causes and indicates that we are getting only partial answers or explanations.

While these are legitimate concerns, these and other critiques of the proximate–ultimate distinction came mostly from those working on general evolutionary questions (not those bringing evolutionary approaches to cancer). Evolutionary biologists have been concerned with whether evolutionary theory needs to be extended to include developmental factors and other causes typically categorized as proximate (Laland et al. 2014). But little to no critique of the distinction has come from developmental biologists. Do studies typically categorized as proximate causation (such as developmental biology) need to consider studies typically categorized as ultimate causation (such as evolutionary biology)? This is a more applicable question in the context of cancer translational research, where much of the research would be categorized as proximate.

The molecular biomarker approach is predominant (by the amount of money allocated, number of research programs, papers, etc.) in the methodologies of developmental biology. Although discovering molecular mechanisms is essential for knowing where to intervene on individuals with cancer, an evolutionary perspective is currently most helpful for developing public health initiatives for communities and populations. That is, an evolutionary perspective helps inform when to start screening and assessing individuals within a population.^10^ But simply identifying or acknowledging the proximate–ultimate distinction does not facilitate bringing together the causal frameworks of the two approaches. The approaches are complementary to each other, but just having both side-by-side is not enough. That is, there are probably important factors or dynamics that are being missed (e.g., reciprocal causation) by not using a framework that considers proximate and ultimate causation together. The standard molecular biomarker and evolutionary biology approaches do not fully capture the complex causal structure, even when considered jointly. We need a perspective on causation that integrates both developmental and evolutionary approaches to cancer in order to better understand cancer and identify effective clinical treatments.

## Overcoming the Proximate–Ultimate Distinction Through Evolutionary Developmental Biology

Integrating proximate and ultimate causal frameworks is not a trivial task; proximate and ultimate causes are typically studied using different methodologies, work on different timescales, and depend on different underlying conceptual foundations. Similar to cancer translational research, evolutionary developmental biology (EvoDevo) aims to explore the connections between the development of individual organisms and their evolutionary transformation to discover causal-mechanistic explanations of individual traits involved in population-level events (Laubichler 2007; Hamilton 2009). Thus I propose turning to EvoDevo for assistance in integrating proximate and ultimate causal frameworks.

EvoDevo can be considered to have two research axes: (1) the evolution of development, or how developmental processes and programs change over time; and (2) the developmental basis of evolution, or how developmental processes and properties affect evolutionary trajectories (Müller 2007; Love 2015). These axes are sometimes separated into “EvoDevo” and “DevoEvo,” respectively (Hall 2000; Gilbert 2003), but here I will consider them together as “EvoDevo.” The discipline of EvoDevo is heterogenous in its methods and questions, pulling from evolution, development, molecular biology, paleontology, and many other disciplines (Müller 2008; Love 2015), and uses key concepts to orient research problems and questions. These concepts include, among others, novelty, modularity, and evolvability (Arthur 2002; Müller 2007; Love 2015).

Though the goals of EvoDevo and cancer translational research are slightly different (namely explanation versus intervention), they have similar desires—integration of population- and individual-level causes, causal-mechanistic explanations of population level events, and understanding how knowledge of individuals and populations inform one another. We can also see how cancer biology can map onto EvoDevo’s research axes: cancer is complex with developmental features evolving over the lifetime of the cancer (EvoDevo), and cancer systems have variation in relevant properties that facilitate change in certain directions (DevoEvo). Thus I suggest cancer translational research follow EvoDevo’s lead and explore how attending to the conceptual issues can help orient research programs.

Cancer researchers do sometimes invoke evolutionary developmental biology but it is often when evolution and development are known to be involved (Kumar et al. 2017) or making use of discovered biology such as details pertaining to certain signaling pathways (Alekseenko et al. 2018). The leaders of EvoDevo have identified cancer as a place where EvoDevo could be helpful in the future, but, again, they make use of the progress in biological discovery by connecting cancer with important developmental pathways and stem cells (Moczek et al. 2015). The use of EvoDevo’s approach and conceptual issues remains untouched but potentially powerful. Here I will apply a notion of modularity and then a related concept of evolvability. In both cases, I will discuss how the concept is used in EvoDevo and then how it could be applied to cancer translational research. I use modularity to show how it is not just the general concept that I find helpful for cancer translational research, but specifically the application of EvoDevo’s version of modularity. The discussion applying evolvability to cancer research is briefer as it is the concept itself that I apply rather than the history of the concept as well.

### Modules and Modularity in EvoDevo

One way EvoDevo has integrated evolution and development is through the notion of modularity (von Dassow and Munro 1999). There are many definitions of modularity but a module is generally understood to be an entity or part that is discrete or *autonomous* in some ways but also *integrated* within a larger whole (usually the rest of the organism) in other ways (Wagner et al. 2007). Modules can be processes or structures (Raff 1996), and be instantiated at any hierarchical level. For example, fragments of *cis*-regulatory DNA (Arnone and Davidson 1997), morphogenic fields, signal transduction pathways, gene regulatory networks (Gilbert and Bolker 2001), and leaf primordia (Gass and Bolker 2003) can all be modules. Furthermore, organisms themselves can be considered modules of higher-level individuals, such as superorganisms (Schlosser and Wagner 2004). Thus, modules can be embedded in higher-order modules (Schlosser 2004).

Modularity involves part-whole relationships, but evolutionary biologists and developmental biologists have thought about those relationships differently. Evolutionary biologists consider modules to be dissociable subunits or parts of a larger system, typically the adult organism (Gass and Bolker 2003). Evolutionary modules are parts autonomous enough to change without appreciably changing other aspects of the organism. Rasmus Winther (2005) calls this a partitioning strategy, where you can understand the whole as a sum of the parts. On the other hand, developmental biologists refer to modules as collectives of entities and processes that act in some unified way to perform a function (Bolker 2000). In this view, a strategy of articulation is used when the relationship or interactions between parts is just as important, if not more, than the parts themselves (Winther 2005). In sum, for evolutionary approaches, the autonomy of a module is usually foregrounded, whereas in developmental approaches, the interactivity or integration between modules is more important.

Regardless of whether autonomy or integration is foregrounded, biologists still recognize that invoking modules and modularity requires being concerned with both autonomy and integration. One way that EvoDevo combines the evolutionary and developmental approaches to modules is that evolutionary modules are the phenotypes that result from particular developmental modules (Gass and Bolker 2003). However, there is not a one-to-one relationship between developmental modules and resulting evolutionary modules (Bolker 2000; Schlosser and Wagner 2004); multiple modules of the same type can be used in a single pathway or process, or modules can overlap by sharing elements (Schlosser 2004). Thus, what is critical for these EvoDevo researchers in bridging the developmental and evolutionary approaches is how modules are individuated and interact with one another. Once meaningful modules are identified, the interactions between them become the focus because it is the *changes in interactions* that result in phenotypic differences and therefore evolutionary change.^11^

This EvoDevo approach to modularity combines evolutionary and developmental approaches to modules and modularity in a particular way (evolutionary modules are the products of developmental modules). By doing this, the EvoDevo researchers shift attention away from proximate and ultimate causation because they can no longer consider the proximate causes (developmental modules) separately from the ultimate causes (the variation linked to evolutionary modules). Though EvoDevo is concerned with the identification of modules (as are evolutionary biologists) and how they interact with each other (similar to developmental biologists), it is the *changes in interactions over time* that get foregrounded for EvoDevo because it is those changes that lead to evolutionary change and population-level events.

Using this version of modularity (where the focus is on changes in interactions over time) as a way to integrate different causal frameworks forces us to shift our attention in two main ways. First, as noted, we shift our attention away from investigative strategies that are specific to proximate or ultimate causation, and thereby avoid treating proximate and ultimate causation separately. Second, as I will discuss below, a causal perspective based on this version of modularity inherently requires the consideration of relationships between multiple levels of organization and across different timescales (e.g., developmental and evolutionary). Consideration of multiple levels and different timescales is not new, but adopting this modularity-based perspective provides a rationale for focusing on the *changes in interactions between the levels* and specifically across time. Thus, a modularity-based causal perspective is one that focuses on the changes in levels of integration of an identified part (module) with respect to other parts (including the organism as a whole) and how those changes affect the system of interest over time.

### Modules and Modularity in Cancer Translational Research

The EvoDevo interpretation of modularity presented here was not meant to replace the evolutionary or developmental interpretations with an intermediate notion but rather integrate parts of each interpretation to allow for the discussion of multiple levels and timescales. Similarly, with cancer I do not aim to necessarily replace the biomarker or evolutionary approaches to cancer translational research, but rather provide a framework for interpreting more complex accounts of causation. Without the modularity framework, investigations usually concerned either the individual (for the biomarker approach) or populations (for the evolutionary approach). With a modularity framework in place, we can identify many potential modules in cancer—various molecular pathways, cells, tumors, tissues, the circulatory system, and/or the immune system, for example. Which modules are meaningful depends on the questions being asked or the phenomena of interest. Because the emphasis is on changes in interactions between modules, no level or system is prioritized but discussions of causal importance are still possible because the same structure for understanding causes holds across levels.

For clarity, let’s consider a generic cancer as it progresses from tumorigenesis to metastasis. At various times, different parts (modules) become the focus and sometimes the success of the cancer depends on increased interactions and sometimes decreased interactions. Cells normally interact in specific and controlled ways to form tissues, but a cell can become cancerous when it gains enough autonomy (i.e., it becomes highly modular) to defect from or cheat within the tissue. As that cell reproduces and grows into a tumor, it progressively loses some autonomy and becomes integrated with its descendent cells in the tumor. As the tumor continues to grow, nutrient and waste transport become important and its dependence on angiogenesis (the development of new blood vessels) and interactivity with the circulatory system increases. For the cancer to spread (metastasize), cells use the circulatory system to get to a new location. Once in a distant tissue, it must integrate into the tissue to evade the immune system. Additionally, when treatment is administered (especially immunotherapy), the cancer forms a different, complex iterative relationship with the immune system.

The interactions between modules and the relationships between the parts and whole are constantly changing depending on the tumor microenvironment, as long as the cancer exists. These multilevel changes in relationships across different timescales facilitate and constrain the evolution of the cancer in certain ways, thus affecting any possible cancer-related treatments. For example, if the increased dependence on the circulatory system is necessary for the evolution of the tumor, then decreasing or preventing the integration with the circulatory system would constrain the cancer’s ability to develop and evolve. Likewise, if the cancer’s survival is dependent on autonomy from the immune system, being able to decrease the cancer’s autonomy will be beneficial for the patient.

What this framework shows is that it is not necessarily better for modules to have higher degrees of autonomy or integration. It is sometimes assumed higher degrees of autonomy are beneficial as that allows independent parts to vary without affecting the function of other parts. But what the above example shows is that in some situations it will be beneficial for there to be high degrees of autonomy (e.g., beginning stages of tumorigenesis) and some situations where it will be beneficial to be integrated (e.g., colonizing distant tissues). And, of course, which is better will depend on which point of view gains the benefits (the host/patient or the cancer).

Additionally, knowing which parts of the organism to consider as meaningful parts and wholes (i.e., what should be studied) is only possible if cancer is considered in its temporal aspect. Different modules become important during different stages of cancer progression. During tumorigenesis, the meaningful part might be the cell and the meaningful whole might be the tissue in which that cell resides. And the question to ask relates to what changed such that the cell was able to become more autonomous within that tissue. The answer could be molecular, ecological, environmental, or some combination. Later in tumor growth, one might want to focus on the relationships between the tumor and the circulatory system. How and why is angiogenesis induced? How do those changes affect the evolutionary trajectory of the cancer? Furthermore, during metastasis, it will be important to investigate the relationships between the clusters of cells that are shed from the tumor, the circulatory system through which they travel, the immune system which they must evade, and the new tissues which they ultimately must colonize. How do some cells evade the immune system while others cannot? Why is a cluster of cells more successful at metastasis than single cells? How do metastases succeed in the new tissues such that they integrate enough to evade the immune system but stay autonomous enough to remain cancerous?

The shift the modularity frameworks brings is shifting from identifying that cells are able to defect from and cheat within a tissue to understanding how those cells are able to. Cells defect and cheat more often than cancers arise, so what are the differences between defections when cancers arise and when they do not? As well, millions of cancer cells circulate throughout the circulatory system but never manifest in disease (Massagué and Obenauf 2016). What are the differences between the circulating tumor cells that eventually die and those that will successfully survive to form metastases?

Tracking multilevel causal interactions through the progression of cancer has the potential to open up novel clinical treatment options that were previously hidden in the divide between proximate and ultimate causation. For example, immunotherapy appears to bridge proximate and ultimate perspectives by striving for the precision of the biomarker approach but also taking advantage of the evolving immune system to combat evolving cancerous systems. Researchers want to find proteins that are over-expressed in all and only cancer cells (such as PD-L1; Iwai et al. 2002) across all time points so that the immune system can continue to target and kill the cancer cells regardless of how the cancer evolves or its state of progression. However, if we apply what I have discussed here regarding the complexity of multilevel causal interactions across time, we can see there is still much more work to be done in order to predict and manage the side effects (e.g., autoimmune disorders) and potential complications or failures of immunotherapies (especially regarding metastasis and recurrent tumors). That is, treatments that make use of both proximate and ultimate causal frameworks still fail to predict complications of the treatment. This modularity perspective automatically suggests that changing the relationship (interactions) between the cancer and the immune system will affect the evolution of the cancer as well as the relationship between the immune system and the patient (i.e., increased chances of developing autoimmune disorders). Knowing what else each module of interest interacts with can point researchers towards potential complications as changing one interaction (e.g., increasing the interactions between the cancer and the immune system) is likely to also change other interactions (such as the interaction between the immune system and other parts of the patient).^12^

As this example shows, identifying differences (i.e., the framework used in the biomarker approach) alone is not problematic. In fact, it is necessary. What are the differences that allow for some cheating cells to become cancerous and others to die? These differences make for potential places of intervention. The modularity framework reminds us that these differences need to be fully interpreted and that causes involve multiple integrated levels that need to be taken into consideration.

### Evolvability in EvoDevo

Evolvability was not a concept of concern to developmental biology or much of evolutionary biology (particularly population genetics) but has, at times, been considered a central concept of EvoDevo (Hendrikse et al. 2007; Minelli 2009). Like modularity, evolvability has many definitions. We can consider the study of evolvability as the study of what facilitates evolutionary change (Love 2015). More specifically, evolvability is defined as “an organism’s capacity to generate heritable phenotypic variation” (Kirschner and Gerhart 1998, p. 8420), or “the capacity of a developmental system to evolve” (Hendrikse et al. 2007, p. 394). Despite slight differences in what level generates the variation or evolves, what is important here is the ability to generate heritable variation.

Evolvability and modularity are related, as it is often considered that high degrees of modularity are required for the evolution of complex phenotypes. But evolvability includes other characteristics besides modularity or autonomy. Marc Kirschner and John Gerhart have identified multiple properties of evolvable systems (i.e., systems that facilitate the generation of heritable variation; Gerhart and Kirschner 2003). The most well-known include compartmentation or modularity, weak regulatory linkage, and exploratory behavior (for more properties see Kirschner and Gerhart 1998). Each of these properties (including those not discussed here) reduces dependence on other parts while providing robustness and flexibility.

### Evolvability in Cancer Translational Research

Evolvability is often studied at the molecular and developmental levels so it seems to be a promising way to integrate the molecular biomarker and evolutionary approaches. As well, genetic variation is known to be associated with faster progression, aggressiveness, and persistence of cancers (Maley et al. 2006; Park et al. 2010). Thus, understanding and identifying properties that facilitate the generation of variation will be important to the development of successful treatments and other clinical applications.

Treatments that use evolutionary theory are often changes in the dosing regimen or treating the microenvironment rather than the cells themselves (Gatenby et al. 2009; Enriquez-Navas et al. 2016). These approaches aim to manage the cancer rather than fully eliminate it. Some researchers have asked, though: What if we could target the evolvability instead? What are the characteristics of evolvable systems and can we target them? (Rosenberg and Queitsch 2014; Fitzgerald and Rosenberg 2017). Essentially the project is to target the drivers of evolution, rather than the products.

Genetic variation is usually thought to be the product of genetic instability due to mutations in DNA repair mechanisms or chromosomal rearrangement. A recent study has shown that, across various cell lines and samples, TGF-beta (transforming growth factor-beta) signaling downregulates DNA repair, which leads to genetically diverse populations. Additionally, exposure to TGF-beta seems to be connected to chemotherapy resistance and (thus) increased adaptability (Fitzgerald and Rosenberg 2017; Pal et al. 2017).

Whether the acquired resistance to chemotherapy actually results in more dangerous cells or cancer is still an open and empirical question but theory suggests that exposure to TGF-beta is causally associated with cancer progression and persistence. Can we target parts of the TGF-beta signaling pathway as a way of slowing evolutionary processes present in cancer progression? There are already clinical trials that target this pathway (Herbertz et al. 2015) but how successful these treatments are also depends on what other processes TGF-beta signaling is involved in. Aspects of wound healing, immune responses, and embryo development use TGF-beta signaling (Fitzgerald and Rosenberg 2017; Pal et al. 2017), reminding us that processes and systems are interconnected and have complex relationships. Thus, we might not want to fully knock down TGF-beta, as having genetic diversity is key for the success of other processes vital to healthy adult life. More work is needed to understand under what circumstances targeting evolvability would be beneficial with respect to treating the cancer, while minimizing side effects.

Again, this example shows that the identification of biomarkers is not problematic. This, and other studies, have identified TGF-beta as upregulated in cancer cells, thus making it a biomarker. However, its presence is reinterpreted within the evolutionary framework. This reinterpretation suggests criteria for why we might think targeting certain molecules or proteins might be more successful than others.

## Conclusion

The molecular biomarker and evolutionary biology approaches to cancer translational research use widely accepted methods of investigation and explanation in biology and biomedical research. These approaches lend themselves well to the current scientific culture as they produce quick results for publications and grants, and ultimately give simple and clean explanations that are easy to understand and convey to others, including the general public. However, they only occasionally produce or lead to “successful” interventions. Additionally, these interventions only help a small percentage of the population (see the Herceptin discussion above), or are far from clinical implementation (e.g., adaptive therapy).^13^ Therefore, following Mayr’s suggestion to maintain separate proximate and ultimate causal frameworks does not work for cancer translational research, nor is it necessarily better than having just proximate or just ultimate causal frameworks. This is because cancer translational research requires more than explanation and understanding. For example, knowing that you were more susceptible to a certain kind of cancer because of an upregulated pathway that gives those cells a fitness advantage does not tell one where to intervene in order to prevent or eliminate the cancer. In other words, the evolutionary biology population approach does not isolate difference makers in such a way that is useful for treating patients. Furthermore, oncologists and society alike would prefer interventions that work without first almost killing the patient (in theory, enough radiation or chemotherapy would kill all of the cancer cells, but the number of healthy cells also killed is far greater than zero). If the goal is effective and relatively safe interventions, we need a framework that embraces the causal complexity of cancer biology and transforms proximate and ultimate investigative strategies into a single, more cohesive perspective.^14^

In this article, I proposed a novel perspective on causation in cancer through evolutionary developmental biology. I argued that the standard research programs map onto Ernst Mayr’s proximate–ultimate distinction, but that in the context of cancer translational research, this distinction can be overcome with the help of evolutionary developmental biology’s strategy to integrate proximate (developmental) and ultimate (evolutionary) causes. I first used a version of modularity that shifts the attention away from individual modules to changes in interactions between modules over time. I suggested that identifying which and how interactions change when, for example, cells switch from being non-cancerous to cancerous could be potential points of intervention. I then used the concept of evolvability as a way to integrate cancer translational research’s molecular biomarker and evolutionary approaches. Using an evolvability lens to reinterpret identified biomarkers that differentiate the healthy from unhealthy helps determine which proteins or molecules might be the best targets. It has been suggested that targeting the properties that drive the generation of variation will be more successful than targeting the products of that variation.

Both of the current predominant approaches suggest that catching and treating cancers early is the best option.^15^ Though shown to be effective in some cases, this is not always possible, nor is it always the best option (see, e.g., Narod et al. 2015; Prasad et al. 2015). Therefore, it is essential to develop treatment regimens for advanced and metastatic cancers. The EvoDevo-based causal perspective described here is one way forward for a research program in cancer translational research that does not deny the vast amounts of existing data, but provides a path to discover and interpret these results more effectively than had previously been done. In addition, this perspective promises to foster research into novel clinical applications.

## Footnotes

1 Though precision medicine and personalized medicine are different projects, there is much overlap between the two and the term “precision medicine” is replacing the use of “personalized medicine” (Katsnelson 2013).

2 Imatinib (Gleevec), used to treat chronic myeloid leukemia (CML), is by far the most successful example of a targeted cancer therapy. However, CML is caused by a single chromosomal rearrangement, which means its causal dynamics are not widely applicable to other cancers.

3 Breast cancers are classified as HER2 + when there is an overexpression of the membrane-bound HER2 (Human Epidermal Growth Factor Receptor type 2) protein. HER2 + breast cancers comprise 20–30% of invasive breast carcinomas, and these patients have decreased overall survival (Hudis 2007).

4 Trastuzumab is still used to treat metastatic HER2 + breast cancers but in combination with other therapies targeted at stopping HER2 from forming dimers with other ligands (Baselga et al. 2012).

5 This is the reason to use standardized mice. These mice are inbred to be genetically identical and raised in the same environments. This underwrites the assumption that everything is identical, except for the knocked-out gene.

6 Note that what follows is not a critique of Woodward. He simply provides the formal language. Woodward is clear that this is a minimal model and not sufficient, and more substantial versions of the interventionist account can handle critiques similar to those I raise. I am arguing that this is the model that the biomarker approach requires.

7 Adaptive therapy is similar to metronomic therapy in that they both use low dose administration of drugs to reduce toxicity and slow tumor regression, as opposed to conventional high dose treatment plans (Bocci et al. 2008). However, where they differ is that metronomic therapies maintain a fixed schedule and adaptive therapy requires assessing the tumor before making a treatment decision (Gatenby et al. 2009).

8 It is important to be specific about what level one wishes to discuss individuals and populations. For example, as described earlier, one can apply evolutionary approaches to populations of cells or people. Regardless of the level of organization, the Darwinian medicine approach cannot tell us about specific individuals within that population (e.g., individual cells within a tumor or an individual patient in a certain population). It can, perhaps, tell us about a population of cells within a specific individual. Thank you to a reviewer for pointing this out.

9 Organizations like the US National Cancer Institute, the main source of funding for the biomarker approach, are starting to fund more evolutionary approaches. However, the budget for the latter approach is dwarfed by the budget for the identification of biomarkers.

10 Those using an evolutionary approach to cancer want to be able to inform treatments and interventions on individuals but are currently not able to. Their success is currently in informing public health initiatives regarding screening practices.

11 The agnathan-to-gnathostome transition is a good example of this, where heterotopic (spatial) changes in developmental modules resulted in the gnathostome-type oral apparatus in which both the upper and lower jaws are derived from the mandibular arch. This is in contrast to the agnathans where only the lower lip is derived from the mandibular arch and the upper lip is derived from the premandibular module (Kuratani 2009).

12 The relationship between the immune system and the patient is incredibly integrated. Thus, even if the interaction relationship under investigation is between the cancer and the immune system, it should not be surprising that changing that relationship is also going to change the patient’s relationship with their immune system.

13 Adaptive therapy has been used in small pilot clinical trials for treatment of prostate cancer (e.g., clinical trial NCT02415621; Zhang et al. 2017).

14 There are other proposals to overcome the limitations of the biomarker approach. For example, Dumitru Iacobas has proposed a Genomic Fabric Perspective (e.g., Iacobas 2016) that compares transcriptomes of networks within individuals across time. This gives a dynamic perspective of integrated networks, which gets at some properties discussed related to modularity. However, it is unclear how this perspective translates into the clinic (I suspect there are at least limitations related to data storage and analysis). I restricted the perspectives discussed here to those that are more widespread and obviously discuss translational goals. I am also ready to accept that multiple frameworks and perspectives might be useful within cancer translational research given the heterogeneity of cancer (i.e., some approaches work better for some cancers). My proposed approach is just one suggestion that would need to be empirically tested (along with other proposed approaches).

15 One might be tempted to say that using an evolvability lens also points to catching and treating cancers early, but variation can be generated throughout cancer’s progression, including through metastatic phases.
